# Supplementary material for: Emergent tetragonality in a fundamentally orthorhombic material
Source: Sci Adv. 2024 May 23;10(21):eadk3321. doi: 10.1126/sciadv.adk3321 (PMC11114214; doi:10.1126/sciadv.adk3321)
Supplement: Supplementary file 1 — Supplementary Text Figs. S1 to S5 [file sciadv.adk3321_sm.pdf]

Science Advances
AAAS

Supplementary Materials for

Emergent tetragonality in a fundamentally orthorhombic material

Anisha G. Singh *et al.*

Corresponding author: Anisha G. Singh, agsingh@stanford.edu

Sci. Adv. **10**, eadk3321 (2024)
DOI: 10.1126/sciadv.adk3321

This PDF file includes:

Supplementary Text
Figs. S1 to S5

Supplementary Text

Measurement of Strains in the Experiment

In this experimental set-up, a uniaxial stress is applied to the sample platform producing strains $\Delta L_x/L_x$, $\Delta L_y/L_y$, and $\Delta L_z/L_z$ in the platform. L_x is the effective or active length of the platform which for this "bowtie" design has been determined by simulation to be 3.47mm. The capacitive sensor in the strain cell allows for measurement of the displacement between the cell's jaws. For low strains we approximate that this displacement is entirely transmitted to the active (narrow) region of the 'bowtie' platform, giving a measurement of ΔL_x. (In practice though, this transmission is likely between 80-90%). However, once the elastic limit of titanium is exceeded, at approximately 0.2%, the capacitive reading is no longer an accurate determination of ΔL_x since the shape of the platform has plastically deformed creating a substantial offset in the actual strain. Instead for large displacements, ΔL_x can be determined by mounting a strain gauge directly onto the Ti platform and a corrective function can be developed to convert the capacitance reading to the actual strain, as shown in Figure S2. This correction is the primary origin of the x-axis error bars shown in Figure. 4A.

While we report our data in terms of measured platform strain, this is a reasonable approximation for the macroscopic strain experienced by the $ErTe_3$ sample. Simulations of samples studied reveal that close to 90% of the platform strain $\Delta L_x/L_x$ is transmitted to the sample. This transmission can also be verified by direct measurement of the sample lattice parameters with XRD. The width of our samples was chosen such that the transverse strain $\Delta L_z/L_z$ is set by the Poisson ratio of the platform rather than the sample. The sample will experience additional strains however due to differential thermal contraction between it and the platform. Since RTe_3 contracts 5 times faster than titanium, the sample will experience additional tensile strains as it cools. This effect is not reflected in the reported value of strain.

The sample's macroscopic strain is different from its microscopic strain in the CDW state, as discussed in the main text. Above the CDW state, ϵ_{xx} and $\Delta L_x/L_x$ are equivalent. This changes with the presence of domains. While the dimensions of the sample change when the platform is strained, within a single domain ϵ_{xx} is zero and will not become nonzero until the sample is strained into one of the monodomain states. This can be seen clearly in Fig. 2B where there is no change in the lattice parameter in the mixed domain phase.

Determination of T_{CDW}

As discussed in the text, T_{CDW} is determined by taking a temperature derivative of the longitudinal resistivity and then locating the peak in that derivative as demonstrated in Fig. S4. (This data is plotted inverted such that the feature corresponds to a peak rather than a dip). Data sets of both the raw data and derivative are offset for clarity. Additionally, only a representative subset of the extracted data shown in Figure 4A is illustrated here.

Elastoresistivity as a Function of Strain

The data presented in Figure 4C is influenced by the definition of the relative temperature. In Fig. S5, the elastoresistivity data are replotted three different ways. First, as done in Fig. 4C**,** a smooth function is used to model T_{CDW} as a function of ($\Delta L_x/L_x$) producing the most accurate result and appropriate even far from the bicritical point. Second, two lines are used to model T_{CDW} as a function of ($\Delta L_x/L_x$) producing a method that is less accurate over the entire strain range, but presumably is still a good description close to the bicritical point. Finally, T_{CDW} is modeled as a constant, clearly an unphysical choice, but is included to demonstrate how robust

the result is. Comparing Figures S4A and S4B, although the exact shape of the peak in the elastoresistance changes depending on the definition of the relative temperature, the peak feature itself is robust. If T_{CDW} is assumed to be constant (Figure S4C), the peak feature is less pronounced, because the elastoresistivity response grows rapidly at high strains due to the strong enhancement of T_{CDW} which is here neglected. Nevertheless, a peak in the elastoresistivity is still observed close to T_{CDW} even in this case.

<u>Tetragonal Approximation of Mean Field Model at Bicritical Point:</u>

Starting from equation 2 in the main text:

$$\Delta F = r_a|\phi_a|^2 + u_a|\phi_a|^4 + r_c|\phi_c|^2 + u_c|\phi_c|^4 + g|\phi_a|^2|\phi_c|^2 + \lambda_a^{xx}\,\epsilon_{xx}|\phi_a|^2 + \lambda_a^{zz}\,\epsilon_{zz}|\phi_a|^2 + \lambda_c^{xx}\,\epsilon_{xx}|\phi_c|^2 + \lambda_c^{zz}\,\epsilon_{zz}|\phi_c|^2 + \ldots$$

At the bicritical point, the coefficients of the quadratic order parameter terms will go to zero. The expression becomes:

$$\Delta F = u_a|\phi_a|^4 + u_c|\phi_c|^4 + g|\phi_a|^2|\phi_c|^2 + \ldots$$

At this point, we can define a renormalization $\phi_c = \left(\frac{u_a}{u_c}\right)^{\frac{1}{4}} \phi'_c$ such that the free energy expression can be rewritten as

$$\Delta F = u_a|\phi_a|^4 + u_a|\phi'_c|^4 + g'|\phi_a|^2|\phi'_c|^2 + \ldots$$

which is now invariant between switching of ϕ_a and ϕ'_c.

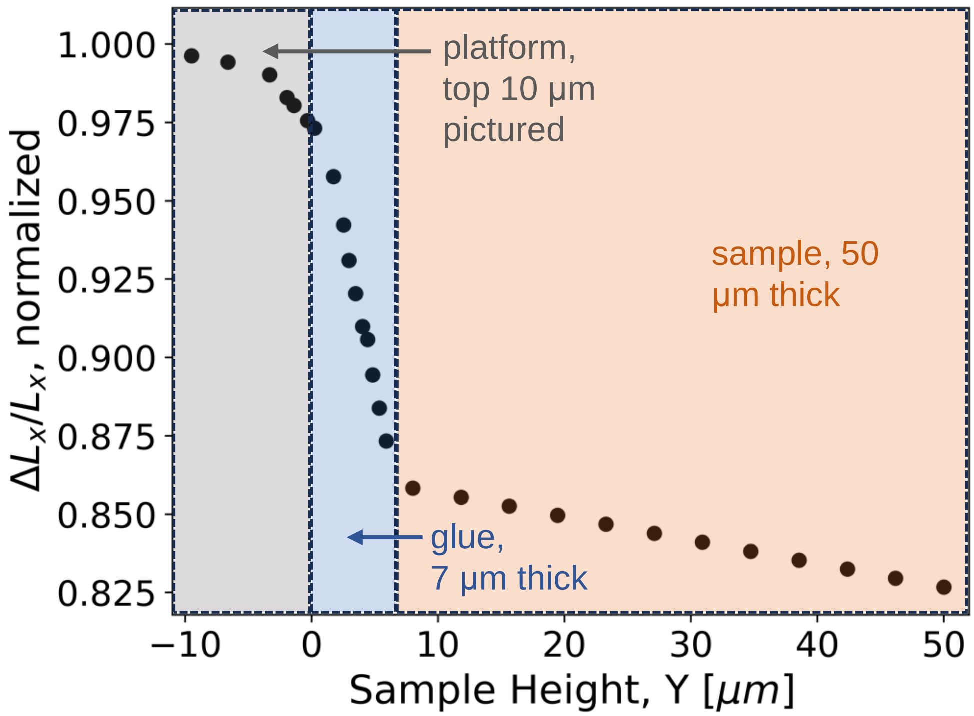

Fig. S1.
Finite element analysis of sample strain across device height: In this simulation, we model the behavior of the device neck with glue and an $ErTe_3$ sample affixed to it with thicknesses defined in the figure. The coordinate axes are defined such that Y= 0µm corresponds to the top surface of the titanium platform. In this simulation, one of the [y, z] faces of the titanium platform is held fixed while a prescribed displacement is then applied along the x- direction. The strains plotted are normalized with respect to this prescribed strain, in order to easily interpret how much the applied strain decays over the height of the device. There is some strain relaxation at the top surface of the platform due to the presence of the sample. The strain transmission falls off mostly through the glue layer, emphasizing the need for thin glue layers. In contrast, over the height of the $ErTe_3$ sample, the applied strain is relatively constant. For the 50 µm thick sample pictured, the variation in strain with sample height is several orders of magnitudes smaller compared to the strain variation applied in the experiment.

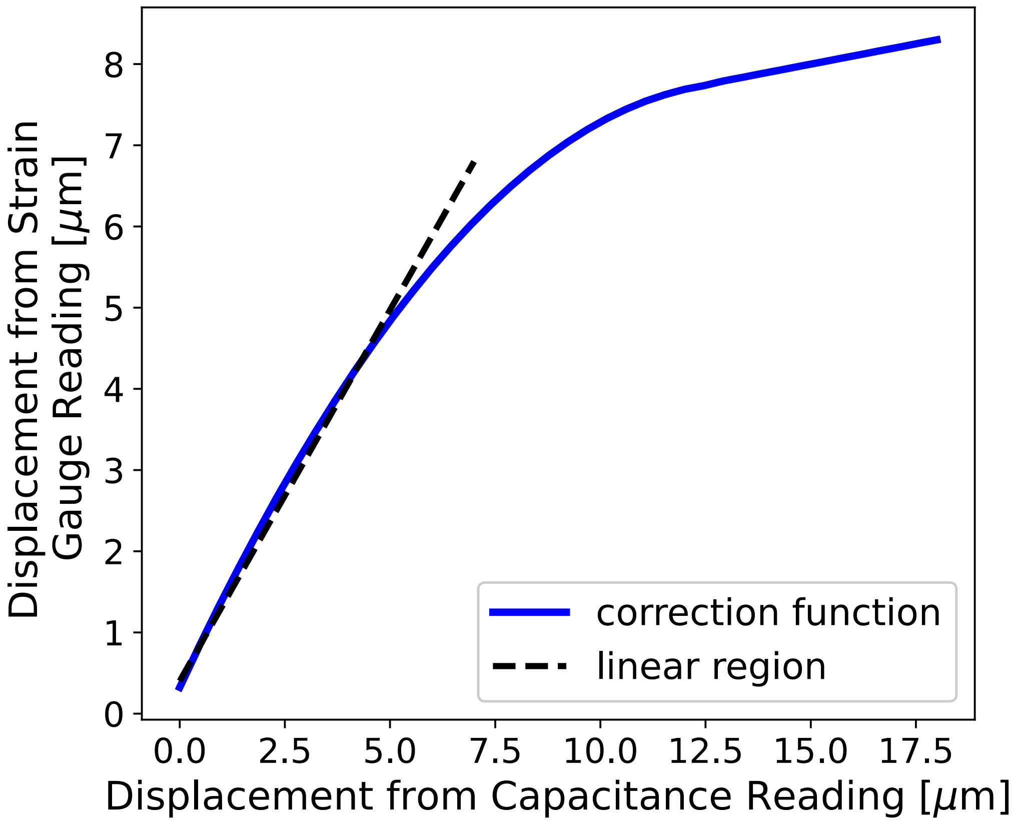

Fig. S2.

Nonlinear response of platform at high offset strains: Displacement measured from strain gauge on platform as a function of displacement measured from capacitive sensor shown in blue. At low strains, this function is linear (dashed black line). The strain gauge reading starts to deviate substantially from the capacitive reading at 7um.

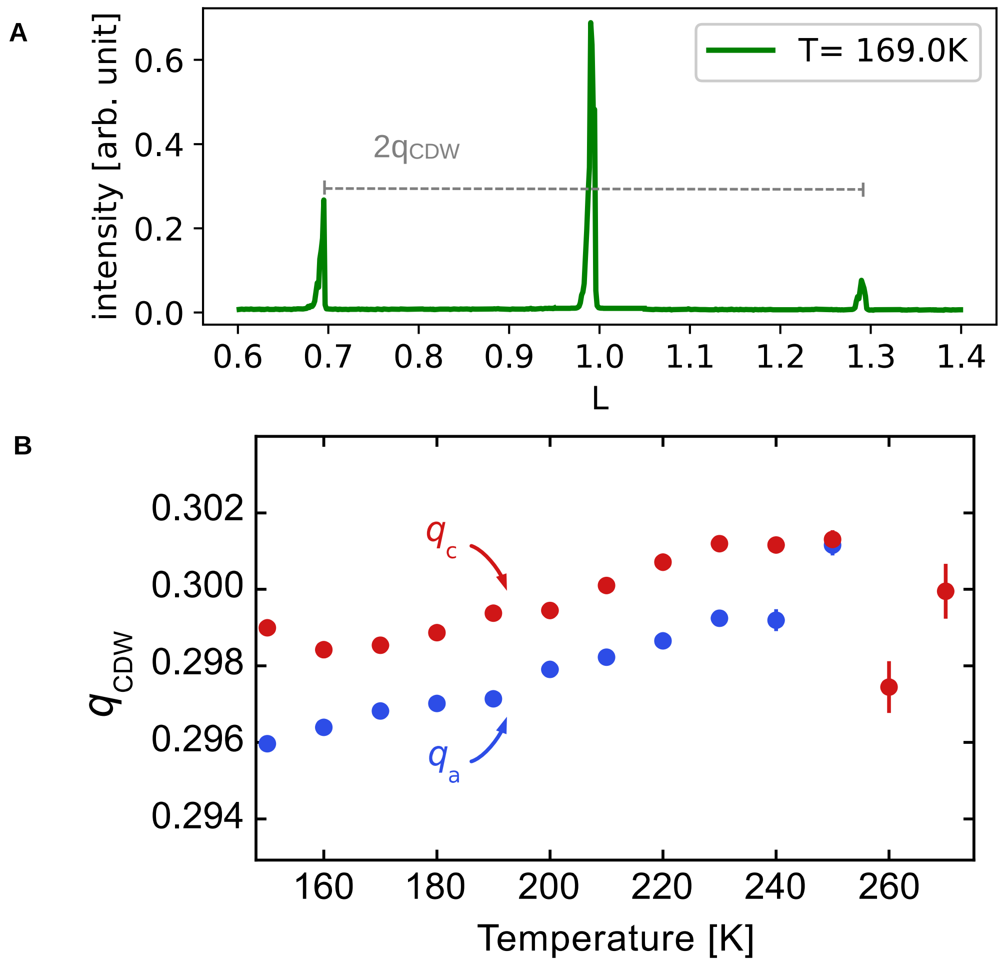

Fig. S3.

q_{CDW} wavevectors as a function of temperature: A) Scan along L direction on (h, k) = 1, 27 plane. By taking the difference between the positions between a pair of superlattice peaks the value of the CDW wavevector can be determined as shown. B) Values of the CDW wavevector observed along the H and L directions specifically, the quantity q_a, plotted in blue, is the observed value of q_{CDW} for the (q_{CDW}, 27, 1) peak in units of a* and the q_c value, plotted in red, corresponds to the value of q_{CDW} observed for the (1, 27 q_{CDW}) peak in units of c*. As an incommensurate order, q_{CDW} can vary with temperature. Both the values of q_a and q_c are similar to the value of q_{CDW}, 0.298 c*, previously observed in $ErTe_3$ (*6*), but notably, the values of q_a and q_c are systematically different from each other. Data presented was collected on a sample mounted on a sapphire rather than titanium platform. In the dataset presented, no bias strain has been applied, but like the sample mounted on titanium, the sample still enters a mixed domain state as a consequence of differential thermal contraction. Since the sample is not manipulated or dynamically strained after bonding to the sapphire, in contrast to the data shown in Figure 2A, the CDW diffraction peaks here are much sharper such that the clear difference in the wave vector can be observed.

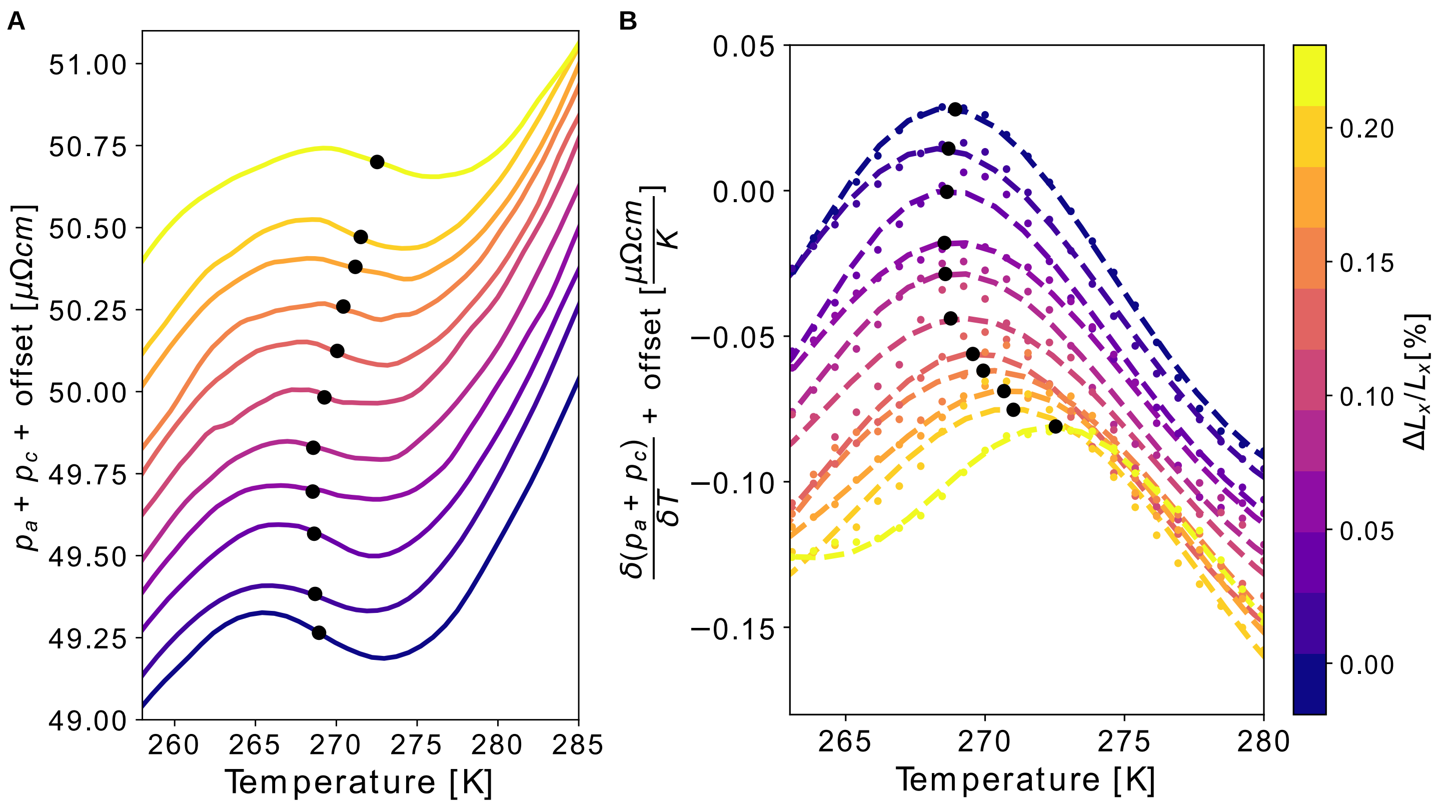

Fig. S4.

Determining *T_{CDW}*: a) $\rho_a + \rho_c$ the longitudinal resistivity plotted as a function of temperature for various offset strains indicated by the color bar. b) Temperature derivative of the longitudinal resistivity used to determine T_{CDW}. Black dots on both plots indicate the value of T_{CDW} for that strain dataset. Identified as the peak in the first derivative, T_{CDW} corresponds to the point at which the resistivity increases the fastest with cooling rather than the higher temperature point at which the resistivity stops decreasing with cooling.

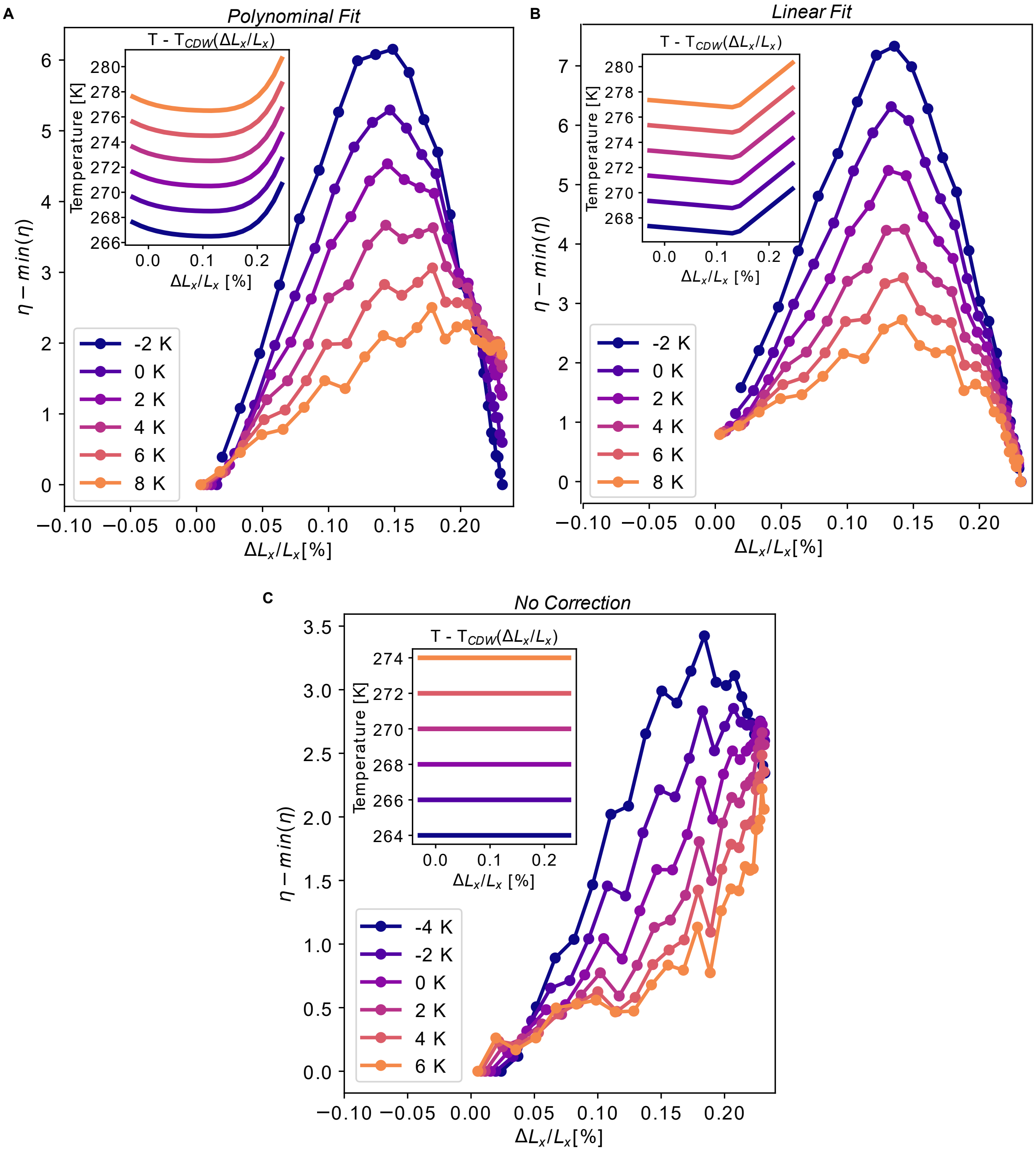

Fig. S5.

Peak in η robust to changes in definition of T_{CDW}: Measured values of η at fixed values of relative temperature for different definitions of relative temperature as defined in the inset of each panel. Each data set is subtracted by its minimum value to facilitate comparison between temperatures.
